# Correlation between Electrode Location and Anxiety Depression of Subthalamic Nucleus Deep Brain Stimulation in Parkinson’s Disease

**DOI:** 10.3390/brainsci12060755

**Published:** 2022-06-08

**Authors:** Feng Zhang, Feng Wang, Yu-Jing Xing, Man-Man Yang, Ji-Wei Wang, Cong-Hui Li, Chun-Lei Han, Shi-Ying Fan, Dong-Mei Gao, Chen Yang, Jian-Guo Zhang, Fan-Gang Meng

**Affiliations:** 1 Department of Neurosurgery, The First Hospital of Hebei Medical University, Shijiazhuang 050031, China; doudougua1979@163.com (F.Z.); xingyujing1986@163.com (Y.-J.X.); 13931893181@163.com (M.-M.Y.); wangjiwei0513@126.com (J.-W.W.); 13363880072@163.com (C.-H.L.); 13513371637@163.com (C.Y.); 2 Departments of Neurosurgery, The First Affiliated Hospital, Zhejiang University School of Medicine, Hangzhou 310006, China; nxwwang@163.com; 3 Departments of Neurosurgery, General Hospital of Ningxia Medical University, Yinchuan 750004, China; 4 Department of Neurosurgery, Beijing Tiantan Hospital, Capital Medical University, Beijing 100070, China; hanchunlei622@163.com (C.-L.H.); fanshiying1907@ccmu.edu.cn (S.-Y.F.); d777ng@126.com (D.-M.G.); zjguo73@126.com (J.-G.Z.); 5 Beijing Neurosurgical Institute, Capital Medical University, Beijing 100070, China; 6 Beijing Key Laboratory of Neurostimulation, Beijing 100070, China; 7 Chinese Institute for Brain Research, Beijing 102206, China

**Keywords:** Parkinson’s disease, subthalamic nucleus, deep brain stimulation, anxiety, depression, volume of tissue activated

## Abstract

Objectives: our group explored the correlation between postoperative coordinates of the electrode contacts, VTA, and anxiety and depression symptoms in Parkinson’s disease (PD) patients after subthalamic nucleus deep brain stimulation (STN-DBS). Methods: STN-DBS was conducted on PD patients (n = 57) for six months with follow-up. Clinical outcomes were explored using the unified Parkinson’s disease rating scale Part III (UPDRS-III), the Hamilton Anxiety Rating Scale (HAM-A), and the Hamilton Depression Rating Scale (HAM-D) before and after surgery. At the Montreal Neurological Institute (MNI), the location of active contacts and the volume of tissue activated (VTA) were calculated. Results: patient evaluations took place preoperatively and follow-ups took place at 1 month, 3 months, and 6 months. The average patient improvement rates for HAM-A and HAM-D scores at the 6-month follow-up were 41.7% [interquartile range (IQR) 34.9%] and 37.5% (IQR 33.4%), respectively (both *p* < 0.001). In medication-off, there were negative correlations between the HAM-A improvement rate and the Z-axis coordinate of the active contact (left side: *r* = −0.308, *p* = 0.020; right side: *r* = −0.390, *p* = 0.003), and negative correlations between the HAM-D improvement rate and the Z-axis coordinate of the active contact (left side: *r* = −0.345, *p* = 0.009; right side: *r* = −0.521, *p* = 0.001). There were positive correlations between the HAM-A and HAM-D scores improvement rate at 6 months after surgery and bilateral VTA in the right STN limbic subregion (HAM-A: *r* = 0.314, *p* = 0.018; HAM-D: *r* = 0.321, *p* = 0.015). Conclusion: bilateral STN-DBS can improve anxiety and depression symptoms in PD patients. The closer the stimulation to the ventral limbic region of the STN, the more significant the improvement in anxiety and depression symptoms of PD patients.

## 1. Introduction

Parkinson’s disease (PD) is a degenerative movement disorder. Deep brain stimulation (DBS) can significantly improve motor symptoms such as tremors in advanced PD patients [1]. Anxiety and depression are very common in PD patients, having a more serious impact on the quality of life compared with motor symptoms. However, the effect of DBS on anxiety and depression in PD patients is unclear. Some previous studies have suggested that DBS can improve anxiety and depression in PD patients [2], whereas others have found the opposite [3]. The most important factors for DBS to improve motor symptoms are accurate electrode implantation and effective postoperative programming. Therefore, the coordinates of the electrode contacts and the volume of tissue activated (VTA) produced by stimulation are critical [4]. The purpose of this study is to analyze the correlation between the improvement rate of anxiety and depression symptoms, the coordinates of the electrode contacts, and volume of tissue activated (VTA).

## 2. Materials and Methods

PD patients (n = 57) with STN-DBS were recruited by our group at the First Affiliated Hospital of Hebei Medical University, the Beijing Tiantan Hospital, and Ningxia Medical University General Hospital between March 2018 and December 2018. We persisted in the tenets of the 1964 Declaration of Helsinki throughout this research. 

### 2.1. Patient Selection

Patients diagnosed with advanced PD according to the UK Parkinson’s Disease Society Brain Bank diagnostic criteria [5] with STN-DBS were selected for inclusion in this study based on evaluation criteria for surgical treatment of PD [6]. Exclusion criteria were severe cognitive impairment, dementia, or psychosis, severe depression (the Hamilton Depression Rating Scale (HAM-D) scores > 29), and severe anxiety (the Hamilton Anxiety Rating Scale (HAM-A) scores ≥ 29). We also excluded those receiving pharmaceutical treatment for their depression or anxiety. We used an acute levodopa challenge test to assess dopaminergic responsiveness and predict the postoperative efficacy of STN-DBS. A predicted improvement rate of >30% was considered an indication for surgery.

### 2.2. Clinical Evaluation

Motor outcomes were assessed using the UPDRS-Ⅲ. Anxiety outcomes were assessed using the HAM-A (14 parts). Depression outcomes were assessed using the HAM-D (24 parts). Quality of life (QOL) was assessed using the 39-item Parkinson’s Disease Questionnaire (PDQ-39). All symptoms of anxiety and depression were assessed both pre- and postoperatively. Postoperative HAM-A scores improvement rate (%) = (preoperative HAM-A scores − postoperative HAM-A scores)/preoperative HAM-A scores × 100%. Postoperative HAM-D scores improvement rate (%) = (preoperative HAM-D scores − postoperative HAM-D scores)/preoperative HAM-D scores × 100%. 

### 2.3. Surgical Procedures

Surgical implantation of the STN-DBS was performed on all patients in this study. In the early morning of surgery, we first fitted the patient with a Leksell stereotactic frame. A framed craniocerebral CT scan was then performed, and CT images were fused with MRI images. The coordinates and entry trajectory of the STN were determined based on the fused image. Electrodes were implanted bilaterally with STN under local anesthesia. Microelectrode recording (MER) was performed intraoperatively. Macrostimulation was used to revalidate target accuracy and side effects. The implantable pulse generator (IPG) was implanted on the same date under general anesthesia. A craniocerebral CT scan was performed immediately after the operation to exclude cerebral hemorrhage, and then the postoperative CT image was fused with the preoperative MR image to determine the position of the electrode.

### 2.4. Postoperative Programming

We turned on the implantable pulse generator (IPG) one month after DBS and performed postoperative programming [7]. We tested all contacts on the electrodes and ultimately chose the best stimulation contacts, which showed the best improvement in patients’ symptoms and the least side effects. At a later date after surgery, parameters were remotely adjusted according to clinical symptoms. The DBS system can provide remote program control for the follow-up of postoperative patients [8]. We first programmed each patient with stimulation parameters: voltage, 1.5–2.0 V; frequency, 130 Hz; pulse width, 60 ms. 

### 2.5. The Coordinates of the Contacts and VTA

The locations of active contacts in STN were located with Lead-DBS software [7,9]. Normalization to the MNI space (Montreal Neurological Institute), and the VTA of STN were calculated in MNI defined by the DISTAL atlas [10,11].

### 2.6. Statistical Analyses

Statistical analysis was performed using SPSS (version 25.0, IBM Corp., Armonk, NY, USA). Raw normally distributed data are reported as mean ± standard deviation (SD). Raw non-normally distributed data are reported as median (M) and interquartile range (IQR). The Friedman test is used for non-normally distributed data. The Pearson correlation analysis was used postoperatively for the anxiety–depression improvement rate and the coordinates of the contacts, VTA. The statistical significance threshold was fixed at *p* < 0.05.

## 3. Results

### 3.1. Patient General Outcomes

PD patients (n = 57): 34 males; 23 females. The LEDD ranged 125–1625 mg/d, the average was 866.3 ± 357.0 mg/d before surgery.

### 3.2. Clinical Outcomes

**Figure 1:**
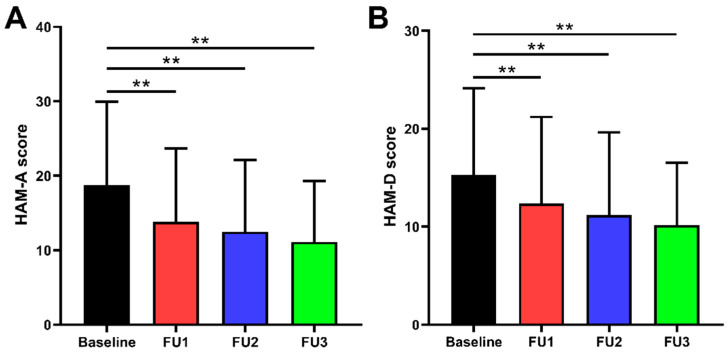
Comparison of preoperative and postoperative HAM-A and HAM-D scores: (**A**) HAM-A scores were improved by 23.5% (IQR 34.9%), 33.3% (IQR 30.9%), and 41.7% (IQR 34.9%) at 1, 3, and 6 months follow-up, respectively. (**B**) HAM-D scores were improved by 20.0% (IQR 33.3%), 31.0% (IQR 32.7%), and 37.5% (IQR 33.4%) at 1, 3, and 6 months follow-up, respectively. ** *p* < 0.001; Baseline = preoperative; FU1 = follow-up 1 month after surgery; FU2 = follow-up 3 months after surgery; FU3 = follow-up 6 months after surgery.

The preoperative improvement rate of UPDRS-Ⅲ scores was 55.4 ± 18.9% (*p* < 0.001), and the reduction rate of LEDD was 40.1 ± 24.3% (*p* < 0.001) in medication-off at the 6-month follow-up. Our comparisons between preoperative and postoperative evaluations are summarized in Table 1 and Figure 1. The improvement rates of HAM-A scores at 1, 3, and 6 months were 23.5% (IQR 34.9%), 33.3% (IQR 30.9%), and 41.7% (IQR 34.9%), respectively (all *p* < 0.001). The improvement rates of HAM-D scores at 1, 3, and 6 months were 20.0% (IQR 33.3%), 31.0% (IQR 32.7%), and 37.5% (IQR 33.4%), respectively (all *p* < 0.001).

### 3.3. Correlation Analysis between the HAM-A, HAM-D Scores Improvement Rate and the Location of Active Contacts

**Figure 2:**
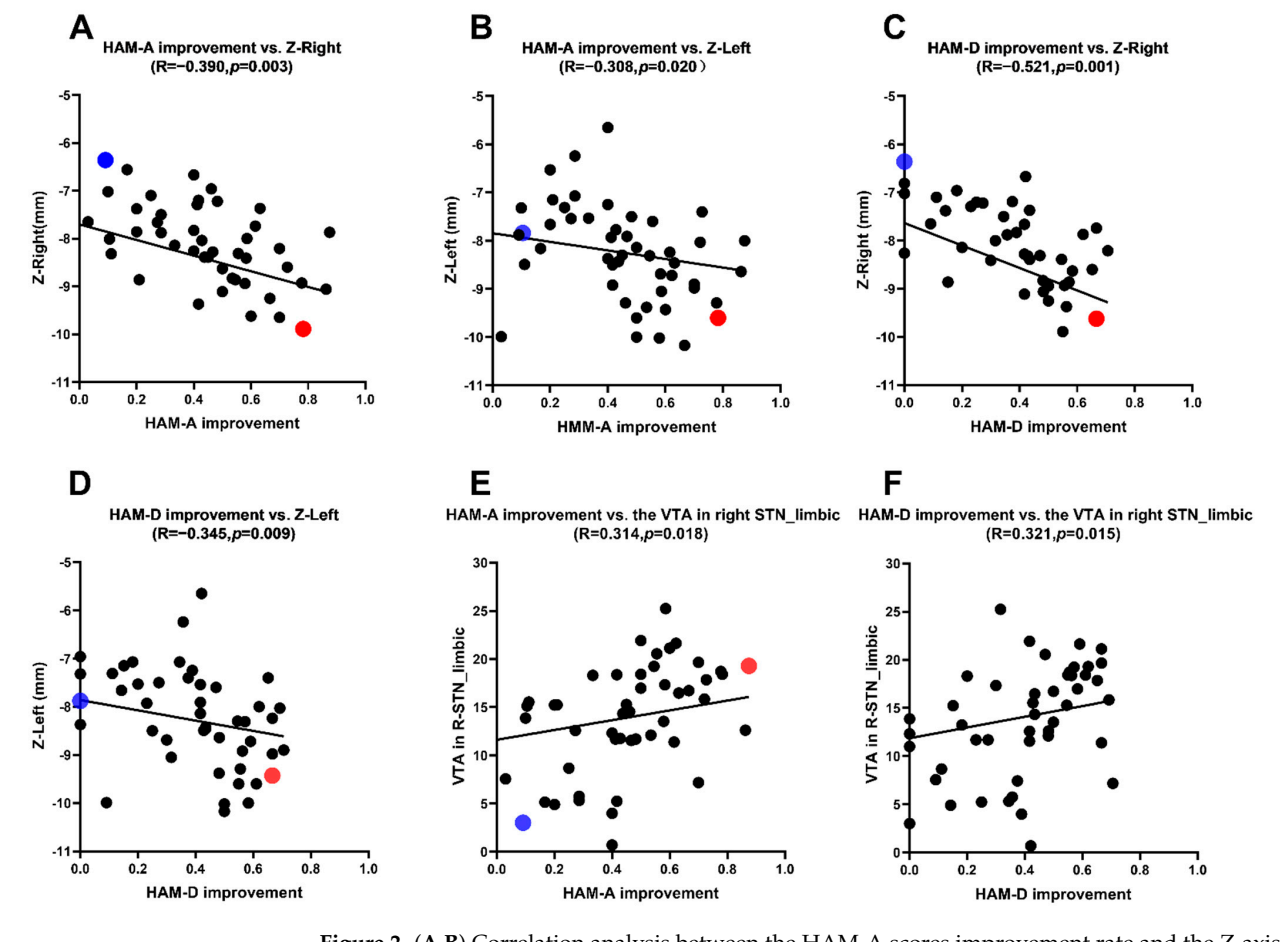
(**A**,**B**) Correlation analysis between the HAM-A scores improvement rate and the Z-axis coordinate. (**C**,**D**) Correlation analysis between the HAM-D scores improvement rate and the Z-axis coordinate. (**E**) Correlation analysis between the HAM-A scores improvement rate and the VTA of limbic STN. (**F**) Correlation analysis between the HAM-D scores improvement rate and the VTA of limbic STN.

**Figure 3:**
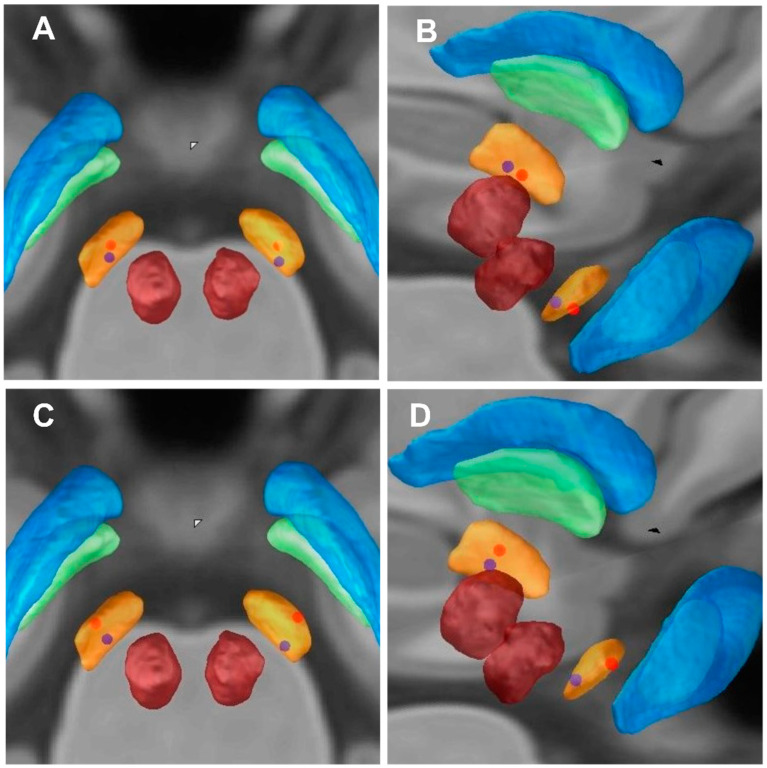
Imaging of electrode contacts. (**A**,**C**) Imaging of electrode contacts (posterior view). (**B**,**D**) Imaging of electrode contacts (right posterior view). (**A**,**B**) the Z-axes of active contact locations of an individual patient (blue dots) with lower HAM-A improvement rate (9.09%); the Z-axes of active contact locations of an individual patient (red dots) with higher HAM-A improvement rate (78.26%). (**C**,**D**) the Z-axes of active contact locations of an individual patient (blue dots) with lower HAM-D improvement rate (0%); the Z-axes of active contact locations of an individual patient (red dots) with higher HAM-D improvement rate (66.67%).

The mean coordinates of active contacts in MNI: left: X = (−11.5 ± 1.1) mm; Y = (−13.5 ± 1.4) mm; Z = (−8.2 ± 1.0) mm; right: X = (12.0 ± 1.1) mm; Y = (−13.5 ± 1.3) mm; Z = (−8.3 ± 1.5) mm. In medication-off there were negative correlations between the HAM-A scores improvement rate and the Z-axis coordinate of the active contact in MNI (left side: *r* = −0.308, *p* = 0.020; right side: *r* = −0.390, *p* = 0.003) and there were negative correlations between the HAM-D scores improvement rate and the Z-axis coordinate of the active contact in MNI (left side: *r* = −0.345, *p* = 0.009; right side: *r* = −0.521, *p* = 0.001) (Table 2). This reveals that the closer the stimulus is to the ventral limbic STN, the higher the improvement of anxiety and depression symptoms in PD patients (Table 2 and Figure 2 and Figure 3).

We selected 2 out of 57 PD patients for anxiety symptoms analysis (blue and red dots patients) (Figure 2A,B and Figure 3A,B). We first analyzed the blue dot patient, located at MNI, the coordinates of electrode active contact: left: x = −11.64 mm, y = −14.97 mm, z = −7.88 mm; right: x = 12.70 mm, y = −14.60 mm, z = −6.63 mm. The HAM-A improvement rate was 9.09%. In contrast, the coordinates of electrode-active contact of the red dot patient: left: x = −11.39 mm, y = −13.20 mm, z = −9.60 mm; right: x = 12.08 mm, y = −13.61 mm, z = −9.89 mm. The HAM-A improvement rate showed the higher improvement rate (78.26%). This reveals that the closer the stimulus is to the ventral limbic STN, the higher the improvement of anxiety symptoms in PD patients. 

Similarly, we selected 2 out of 57 PD patients for depression symptoms analysis (blue and red dot patients) (Figure 2C,D and Figure 3C,D). We first analyzed the blue dot patient, located at MNI, the coordinates of electrode active contact: left: x = −11.64 mm, y = −14.97 mm, z = −7.88 mm; right: x = 12.70 mm, y = −14.60 mm, z = −6.63 mm. The HAM-D improvement rate was 0%. In contrast, for the red dot patient, the coordinates of electrode active contact: left: x = −12.21 mm, y = −14.17 mm, z = −9.43 mm; right: x = 12.14 mm, y = −14.89 mm, z = −9.62 mm. The HAM-D improvement rate was higher (66.67%). This reveals that the closer the stimulus is to the ventral limbic STN, the higher the improvement of depression symptoms in PD patients. 

### 3.4. Correlation Analysis between the HAM-A, HAM-D Scores Improvement Rate and the VTAs

The VTAs in the STN limbic subregion in the MNI space were: left: 14.7 (8.2) mm^3^; right: 14.3 (9.0) m^3^. There were positive correlations between the HAM-A and HAM-D scores improvement rate and the VTAs in the right STN limbic subregion (HAM-A: *r* = 0.314, *p* = 0.018; HAM-D: *r* = 0.321, *p* = 0.015) (see Table 3 and Figure 2E,F).

**Figure 4:**
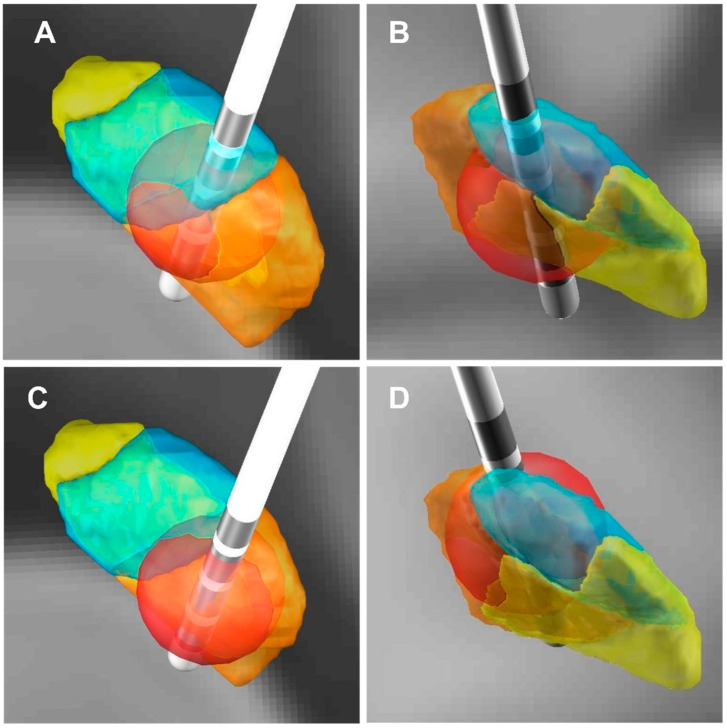
Three-dimensional illustration of VTA and its relationship with the improvement rate of the HAM-A scores. (**A**,**C**) Three-dimensional illustration of VTA (posterior view). (**B**,**D**) Three-dimensional illustration of VTA (front view). (**A**,**B**) The red dot patient (the HAM-A improvement rate: 87.50%), the VTA in the right STN limbic subregion in this patient was 19.29 mm^3^. (**C**,**D**) The blue dot patient (the HAM-A improvement rate: 9.09%), the VTA in the right STN limbic subregion in this patient was 3.01 mm^3^. (The red ball: VTA; The orange area: STN sensorimotor subregion; The blue area: STN associative subregion; The yellow area: STN limbic subregion).

We selected 2 out of 57 PD patients for anxiety symptoms analysis (blue and red dot patients) (Figure 3A,B and Figure 4). For the red dot patient (Figure 3A,B), the VTA in the right STN limbic subregion was 19.29 mm^3^, and the HAM-A improvement rate was 87.50% (Figure 4A,B). In contrast, for the blue dot patient (Figure 3A,B), the VTA in the right STN limbic subregion was 3.01 mm^3^, and the HAM-A improvement rate was 9.09% (Figure 4C,D). This reveals that the higher the VTA in the right STN limbic subregion, the higher the improvement of anxiety symptoms in PD patients. 

## 4. Discussion

Depression is very common in PD patients. About 40–50% of PD patients have depression, with 5–10% developing severe depression [12]. The reported effects of DBS on depression vary widely. Some studies suggest that DBS can aggravate depressive symptoms [3], arguing that the stimulation of brain regions involved in behavior can induce a range of disorders, of which depression and anxiety are symptoms [13]. Others have reported significant improvements in depressive symptoms resulting from DBS [14]. In our finding, the HAM-D scores improvement rate was 37.5% (IQR 33.4%, *p* < 0.001) 6 months after the operation. Although we excluded patients with severe depression, our patients with mild and moderate depression showed significant symptomatic improvements after undergoing STN-DBS. None of the patients in our cohort were receiving pharmacological treatment for anxiety or depression. It has been posited that depressive symptoms may have multiple causes, including the degeneration of the substantia nigra striatum, as well as psychological and environmental factors. Stimulation of STN-DBS electrode and reduction in levodopa after operation may have some causative influence on the increase in depressive symptoms [15,16]. However, we did not observe any exacerbation of depression following STN-DBS in our study. Anxiety is also a frequent secondary symptom of PD, affecting about 40% of PD patients [12]. Previous studies have reported significant improvements in anxiety after STN-DBS [17], which was also supported by our study. We saw an improvement rate in HAM-A scores of 41.7% (IQR 34.9%, *p* < 0.001) at 6-month follow-ups. The improvement of depression after STN-DBS may be related to a number of factors. Neurostimulation of DBS increases dopamine levels in the brain [18], and dopamine is closely related to happiness and well-being. DBS may improve the metabolism of neuronal cells, reduce the hyperresponsiveness of cells, increase the euphoria in PD patients, regulate the related transmitters that trigger depression, change the concentration of inflammatory mediators, and regulate the excitability of astrocytes. It can improve the depressive symptoms of PD patients [19]. Moreover, DBS treatment can improve the movement disorder by inhibiting the frontal lobe, and the patient’s mood and neurological function will also improve after the movement disorder is improved. It is therefore extremely important to assess depressive symptoms multiple times in the perioperative period. GPi targets can be tried in patients with PD and severe depressive symptoms.

Frontal loops are also known as coordination loops. The frontal circuit is involved in cognition, emotion, and behavior [20]. STN can be anatomically divided into three functional subregions [20]: a dorsolateral motor region, an intra-ventral coordination region, and a medial limbic region. These are associated with movement, coordination, and emotion, respectively. On the other hand, many research results show that the basal ganglia are an integral part of the cognitive and emotional circuits of the brain [21,22]. Moreover, the more the electrode contact stimulation is to the ventral side of STN, the better the improvement of anxiety and depression symptoms [23]. According to the functional partition of STN, the STN limbic subregion is ventral medial to the subthalamic nucleus [24], and DBS stimulation of this region may modulate the limbic circuit of the brain, thereby improving neuropsychiatric symptoms such as anxiety and depression to a greater extent. A study showed that [25] neurostimulation had better results in the ventral STN than dorsal STN for anxiety [26], the reason being that the limbic subregion in the functional partition of STN is located in the ventral subregions of STN [22]. In our study at MNI, the HAM-A and HAM-D improvement rate was negatively correlated with the Z-axis coordinate of the active contact. This indicates that the closer to the ventral limbic STN, the higher the HAM-A, HAM-D improvement rate. Therefore, the higher the improvement of anxiety and depression symptoms in PD patients. The more the VTA in the right STN limbic subregion, the higher the HAM-A and HAM-D improvement rate. This study is similar to previous reports [23]. We think that the improvement of anxiety and depression in PD patients after STN-DBS treatment was associated with stimulation of the ventral STN. Furthermore, we infer that there is no obvious boundary between the subregions of STN; subregions of STN are not strictly anatomic subregions, they have functional overlaps. Despite the statistical significance, there is still a fair bit of scatter in Figure 2. Clearly, the impact of stimulation on mood is complicated, and there are likely a number of confounds not accounted for in terms of region of stimulation and impact on mood. Some studies have shown that DBS can worsen symptoms of anxiety and depression in patients with PD [3], however, our study came to the opposite conclusion that bilateral STN-DBS can improve anxiety and depression symptoms in PD patients, cautioning that further scrutiny is still merited, particularly in patients who have underlying mood disorders.

### Limitations

This study had three main limitations. Firstly, the evaluation tools used (the HAM-A and HAM-D questionnaires) allow a certain degree of subjectivity both in the evaluation of patient anxiety and depression and in the interpretation of the associated scales. This may cause different assessors to obtain different results. Secondly, the final postoperative follow-up was at 6 months, so we were unable to assess the long-range impacts of DBS on anxiety and depression. 

## 5. Conclusions

Bilateral STN-DBS can improve anxiety and depression symptoms in PD patients. The closer the stimulation is to the ventral limbic region of the STN, the more significant the improvement in anxiety and depression symptoms in PD patients.

## Figures and Tables

**Table 1:** HAM-A and HAM-D scores after STN-DBS [M (IQR)].

	Preoperative	Postoperative			Total *p*	χ2	*p*_1_	*p*_2_	*p*_3_
		1 Month	3 Months	6 Months					
HAM-A (0–56)	16 (14)	11 (11)	9 (10)	11 (11)	<0.001	60.464	<0.001	<0.001	<0.001
HAM-D (0–68)	14 (13)	9 (9)	9 (8)	9 (9)	<0.001	45.592	<0.001	<0.001	<0.001

HAM-A, Hamilton Anxiety Rating Scale; HAM-D, Hamilton Depression Rating Scale. The *p*_1_, *p*_2_, and *p*_3_ values are the comparisons between the preoperative scores and the scores 1, 3, and 6 months postoperative, respectively.

**Table 2:** HAM-A, HAM-D scores improvement rate and active contact locations.

	HAM-A Improvement Rate		HAM-D Improvement Rate	
	r	*p*	r	*p*
right				
X	0.017	0.899	−0.063	0.642
Y	0.003	0.985	0.032	0.811
Z	−0.390	0.003	−0.521	0.001
left				
X	0.237	0.076	0.210	0.117
Y	0.027	0.842	−0.078	0.566
Z	−0.308	0.020	−0.345	0.009

**Table 3:** Correlation between HAM-A, HAM-D scores improvement rate and the VTAs.

	HAM-A Improvement		HAM-D Improvement	
	r	*p*	r	*p*
right STN				
motor subregion	−0.027	0.839	−0.085	0.531
associative subregion	−0.176	0.190	−0.189	0.159
limbic subregion	0.314	0.018	0.321	0.015
left STN				
motor subregion	−0.078	0.566	0.163	0.226
associative subregion	0.086	0.524	0.042	0.755
limbic subregion	0.174	0.196	0.154	0.254

## Data Availability

The data of this study are available from the first author.

## References

[1] Lu J., Feng Z., Shi X., Jiang L., Hao Y. (2020). Correlation between programmed stimulation parameters and their efficacy after deep brain electrode implantation for Parkinson’s disease. J. Neurorestoratol..

[2] Wichmann T., DeLong M.R. (2016). Deep brain stimulation for movement disorders of basal Ganglia origin: Restoring function or functionality?. Neurotherapeutics.

[3] Birchall E.L., Walker H.C., Cutter G., Guthrie S., Joop A., Memon R.A., Watts R.L., Standaert D.G., Amara A.W. (2017). The effect of unilateral subthalamic nucleus deep brain stimulation on depression in Parkinson’s disease. Brain Stimul..

[4] Zhang F., Wang F., Li W., Wang N., Han C., Fan S., Li P., Xu L., Zhang J., Meng F. (2021). Relationship between electrode position of deep brain stimulation and motor symptoms of Parkinson’s disease. BMC Neurol..

[5] Berg D., Lang A.E., Postuma R.B., Maetzler W., Deuschl G., Gasser T., Siderowf A., Schapira A.H., Oertel W., Obeso J.A. (2013). Changing the research criteria for the diagnosis of Parkinson’s disease: Obstacles and opportunities. Lancet Neurol..

[6] Zach H., Walter U., Liepelt-Scarfone I., Maetzler W. (2017). Diagnostics of clinical and prodromal idiopathic Parkinson’s disease: New criteria. Nervenarzt.

[7] Horn A., Reich M., Vorwerk J., Li N., Wenzel G., Fang Q., Schmitz-Hübsch T., Nickl R., Kupsch A., Volkmann J. (2017). Connectivity Predicts deep brain stimulation outcome in Parkinson disease. Ann. Neurol..

[8] Chen Y., Hao H., Chen H., Li L. (2015). The study on a telemedicine interaction mode for Deep Brain Stimulation postoperative follow-up. Annu. Int. Conf. IEEE Eng. Med. Biol. Soc..

[9] Husch A., Petersen M.V., Gemmar P., Goncalves J., Hertel F. (2018). PaCER—A fully automated method for electrode trajectory and contact reconstruction in deep brain stimulation. Neuroimage Clin..

[10] Ewert S., Plettig P., Li N., Chakravarty M.M., Collins D.L., Herrington T.M., Kühn A.A., Horn A. (2018). Toward defining deep brain stimulation targets in MNI space: A subcortical atlas based on multimodal MRI, histology and structural connectivity. Neuroimage.

[11] Pauli W.M., Nili A.N., Tyszka J.M. (2018). A high-resolution probabilistic in vivo atlas of human subcortical brain nuclei. Sci. Data.

[12] Dulski J., Schinwelski M., Konkel A., Grabowski K., Libionka W., Wąż P., Sitek E.J., Sławek J. (2019). The impact of subthalamic deep brain stimulation on sleep and other non-motor symptoms in Parkinson’s disease. Parkinsonism Relat. Disord..

[13] Jaafari N., Giré P., Houeto J.L. (2009). Deep brain stimulation, Parkinson’s disease and neuropsychiatric complications. Presse Med..

[14] Castelli L., Perozzo P., Zibetti M., Crivelli B., Morabito U., Lanotte M., Cossa F., Bergamasco B., Lopiano L. (2006). Chronic deep brain stimulation of the subthalamic nucleus for Parkinson’s disease: Effects on cognition, mood, anxiety and personality traits. Eur. Neurol..

[15] Bronstein J.M., Tagliati M., Alterman R.L., Lozano A.M., Volkmann J., Stefani A., Horak F.B., Okun M.S., Foote K.D., Krack P. (2011). Deep brain stimulation for Parkinson disease: An expert consensus and review of key issues. Arch. Neurol..

[16] Follett K.A., Weaver F.M., Stern M., Hur K., Harris C.L., Luo P., Marks W.J., Rothlind J., Sagher O., Moy C. (2010). Pallidal versus subthalamic deep-brain stimulation for Parkinson’s disease. N. Engl. J. Med..

[17] McDonald L.M., Page D., Wilkinson L., Jahanshahi M. (2012). Deep brain stimulation of the subthalamic nucleus improves sense of well-being in Parkinson’s disease. Mov. Disord..

[18] Blomstedt P., Hariz M.I., Lees A., Silberstein P., Limousin P., Yelnik J., Agid Y. (2008). Acute severe depression induced by intraoperative stimulation of the substantia nigra: A case report. Parkinsonism Relat. Disord..

[19] Etiévant A., Lucas G., Dkhissi-Benyahya O., Haddjeri N. (2016). The Role of Astroglia in the Antidepressant Action of Deep Brain Stimulation. Front. Cell. Neurosci..

[20] Wang X., Chang C., Geng N., Li N., Wang J., Ma J., Xue W., Zhao W., Wu H., Wang P. (2009). Long-term effects of bilateral deep brain stimulation of the subthalamic nucleus on depression in patients with Parkinson’s disease. Parkinsonism Relat. Disord..

[21] Frank M.J., Samanta J., Moustafa A.A., Sherman S.J. (2007). Hold your horses: Impulsivity, deep brain stimulation and medication in Parkinsonism. Science.

[22] Krack P., Hariz M.I., Baunez C., Guridi J., Obeso J.A. (2010). Deep brain stimulation: From neurology to psychiatry?. Trends Neuro. Sci..

[23] York M.K., Wilde E.A., Simpson R.A. (2009). Relationship between neuropsychological outcome and DBS surgical trajectory and electrode location. J. Neuro. Sci..

[24] Brunenberg E.J., Moeskops P., Backes W.H., Pollo C., Cammoun L., Vilanova A., Janssen M.L., Visser-Vandewalle V.E., ter Haar Romeny B.M., Thiran J.P. (2012). Structural and resting state functional connectivity of the subthalamic nucleus: Identification of motor STN parts and the hyperdirect pathway. PLoS ONE.

[25] Dafsari H.S., Petry-Schmelzer J.N., Ray-Chaudhuri K., Ashkan K., Weis L., Dembek T.A., Samuel M., Rizos A., Silverdale M., Barbe M.T. (2018). Non-motor outcomes of subthalamic stimulation in Parkinson’s disease depend on location of active contacts. Brain Stimil..

[26] Gourisankar A., Eisenstein S.A., Trapp N.T., Koller J.M., Campbell M.C., Ushe M., Perlmutter J.S., Hershey T., Black K.J. (2018). Mapping movement, mood, motivation and mentation in the subthalamic nucleus. R. Soc. Open Sci..

